# Mediterranean Diet and its Benefits on Health and Mental Health: A Literature Review

**DOI:** 10.2174/1745017902016010156

**Published:** 2020-07-30

**Authors:** Antonio Ventriglio, Federica Sancassiani, Maria Paola Contu, Mariateresa Latorre, Melanie Di Salvatore, Michele Fornaro, Dinesh Bhugra

**Affiliations:** 1 Department of Clinical and Experimental Medicine, University of Foggia, Foggia, Italy; 2 Dipartimento di Scienze Mediche e Sanità Pubblica, Università degli Studi di Cagliari, Cagliari, Italy; 3 Dipartimento di Scienze Chirurgiche, Università degli Studi di Cagliari, Cagliari, Italy; 4 Department of Neuroscience, Reproductive Science and Odontostomatology, School of Medicine 'Federico II' Naples, Naples, Italy.; 5 Institute of Psychiatry, King’s College, London, UK

**Keywords:** Mediterranean Diet, Health, Mental Health, Depression, Anxiety, Cancer

## Abstract

Mediterranean Diet (MD) is currently considered one of the most healthy dietary models worldwide. It is generally based on the daily intake of fruit and vegetables, whole grains, legumes, nuts, fish, white meats, and olive oil. It may also include moderate consumption of fermented dairy products, a low intake of red meat, and red/white wine during the main course. Even if the effect of MD on cancer prevention as well as on human metabolic and cardiovascular balance has been discussed, including the quality of life of the exposed population, the putative effects on mental health are still not properly investigated. This narrative review reports on some emerging pieces of evidence on the possible impact of MD on general health and the outcome of psychiatric disorders (*e.g*., major depression, anxiety) and encourages further studies to test the benefits of healthy food selection on the health of the general population.

## 1 INTRODUCTION

Mediterranean Diet (MD) is currently considered one of the most healthy dietary models worldwide [1]. It was described, at first, by Ancel Keys as a dietary regimen based on low quantities of fat oils and high levels of vegetable oils, widespread in Greece and South of Italy in the ‘60s [2, 3]. The Greek Dietary Guidelines (1999) [4], the Mediterranean Diet Foundation (2011) [5], and Oldway’s Preservation and Trust (2009) [6] proposed three Mediterranean Diet Pyramids recommending respectively: *a)* olive oil, vegetables, fish, bread and cereals > 6 serves/daily; eggs, legumes and nuts 3-4 serves/daily; *b)* olive oil at every meal, vegetables, fruits, fish, legumes and cereals ≥ 2 serves/daily; and *c)* olive oil, vegetables, fish, legumes, cereal and bread at every meal. MD may also include moderate consumption of fermented dairy products, and a low intake of red meat and moderate consumption of wine during the main courses [2].

MD, from a nutritional point of view, is low in saturated fats and animal proteins, rich in antioxidants, fibers and monounsaturated fats and shows an adequate balance of omega-6 / omega-3 fatty acids. Therefore, benefits on human health may be explained on the high intake of antioxidants, fibers, monounsaturated and omega-3 fatty acids, phytosterols and probiotics [1, 2].

Several studies have described the food consumption patterns and related health consequences around the world. At the end of ‘50s, a large study named The Seven Countries’ study (which included Italy, Finland, Greece, United States, the Netherlands, Yougoslavia, Japan) confirmed that MD was adopted in Greece and Italy, whereas in some Nordic countries, the consumption of sugars, fats, milk and potatoes was very high. The United States reported high consumption of sugars, fruit, meat, while Japan reported a higher consumption of fish and rice [7]. The PREDIMED (PREvención con DIeta MEDiterránea) multicenter study assessed and described the long-term effects of MD on cardiovascular disease and other clinical conditions [8- 11]. It has been launched in 2013 [9] and randomized 7447 patients to MD with supplementation of olive oil or nuts *versus* control diet. A large amount of study- reports have been published over the years with lights and shadows about the validity of sampling since MD was differently considered among cohorts of the study [10] and control diet was not specifically described for comparisons. A second- step- study named PREDIMED-Plus has been conducted to evaluate the impact of lifestyle intervention and energy-restricted MD on the primary prevention of cardiovascular disease among 6874 subjects recruited in 23 centers and hospitals in Spain. These combined interventions, energy-restricted MD with higher intake of polyphenols and physical activity, were significantly associated with an improvement of compounds of metabolic syndrome among subjects involved such as inflammatory biomarkers and blood cholesterol and triglycerides [12- 15]. This large trial has also demonstrated that MD may improve the general quality of life of subjects and physical activity may have benefits on quality of life and cognitive functioning [16, 17].

It is of interest that a new large study, named PREDI-DEP, has been launched to assess the employment of Mediterranean Diet (MD) supplemented with extra virgin olive oil or nuts in preventing the risk of relapse of unipolar depression over two years of clinical follow-up [18]. This may add to the evidence that MD moderates the association between multimorbidity and depressive symptoms among major depression patients as reported by an Italian study group in 2020 [19].

Also, it is of note that a healthy dietary regimen and physical exercise should be generally recommended for mentally ill patients to improve the outcome of illness and reduce mortality for metabolic syndrome and cardiovascular diseases mostly related to unhealthy lifestyle, physical comorbidities and psychotropic treatments [20-23].

This narrative review reports on available evidence on the possible impact of MD on health and mental health and encourages further studies to test the benefits of healthy food selection and healthy lifestyle on the general population.

## 2 METHODS

Scientific literature research was performed *via* Medline (PubMed version) employing the combination of keywords “Mediterranean Diet” AND: “Health”, “Mental Health”, “Depression”, “Anxiety”. We obtained and analyzed 65 studies of which 27 were selected because they met the following inclusion criteria: full-text articles, cohort, case-control or Randomized Clinical Trial study projects. We excluded articles published in languages ​​other than English, design studies other than RCT (n= 13), those focused on wine and drinks (n= 2), on adherence to a Mediterranean diet only (n= 11) and study protocol description only (n= 6) (see PRISMA [24] flow diagram; Fig. (**1**). Twenty-seven articles were finally selected and analyzed for this report.

## 3 RESULTS

We focused the report on 27 articles as shown in the PRIMA diagram [25- 51]; 13 of these studies were carried out in Spain [26-28, 33, 35, 38, 41, 44 - 46, 48, 49, 51], 3 studies were carried out in the USA [25, 31, 47], 3 studies were carried out in Italy [30, 36, 40], 4 studies were carried out in other European Union countries [32, 34, 39, 50] and 4 studies were carried out in Australia [29, 37, 42, 43].

### 3.1 Characteristics of Samples in the Studies

Collectively, the studies involved 16,584 participants including 7447 from the PREDIMED- Trial reports [41, 44, 45, 48, 51]. Of all subjects involved in the studies, 4092 represented a healthy population, of which 2086 were children. We divided the studies into 9 groups based on different aims of the studies and population: *healthy or young subjects, post-menopausal women, elderly subjects, pregnant women, depressive disorder patients, type-2 diabetes mellitus, cardiovascular diseases, cancer, and metabolic syndrome* (Table **1**). One study [38] examined a population of 874 pregnant women to describe gestational diabetes and dietary interventions, one study considered postmenopausal women [40], and two studies considered elderly population [34, 42]. Regarding the evaluation of the impact of MD on specific health conditions, only 2 Australian studies considered the impact of diet on mental health including a total of 219 participants [37, 43], 3 studies described the effects of MD on patients with Type 2- Diabetes Mellitus, 9 studies involved samples of people affected or at risk of cardiovascular disease, 2 studies reported the effect of diet on subject affected or at risk of cancer, 2 studies reported about the impact of MD on metabolic syndrome (Table **1**).

We also considered the mean ± standard deviation of the age of subjects sampled in the 27 studies. Studies involving “Healthy Subjects” reported high heterogeneity in age. Other studies reporting on physical morbidities (Type 2 Diabetes, Cardiovascular Diseases, Cancer, Metabolic Syndrome) involved people with age ranging from 50 and 65. Pregnant women group reported 32.7 years old whereas Depressive Disorder patients reported 44.32 years old (Table **2**).

Since all the studies evaluated the impact of diet on the nutrition state of studies’ subjects, we have gathered data about Body Mass Index (BMI) as shown in Table **3**. Three studies didn’t report the BMI of their sample [37, 38, 46]. A significant improvement of BMI, as an outcome measure of being on MD, was found in four studies (all *p*≤0.01*;* [30, 39, 44, 48]).

### 3.2 Studies’ Design

The heterogeneity between each study's definition of dietary variables and the multiplicity of applications of MD in the different research works precluded any formal meta-analysis. The studies had different aims related to the type of population recruited. As shown in Table **1**, most of the studies evaluated the impact of diet on metabolism (including diabetes) and cardiovascular outcomes. Two studied focused on the impact of diet on samples affected or at risk of cancer and only 4 studies focused on detecting “quality of life” or improvements in depressive symptoms (2 studies). The data were mainly derived from RCT studies with or without a parallel-group design. 2 studies had a crossover RCT design [28, 29]. In most of the cases, there was an intervention group following MD and a control group with its usual diet. In 9 studies [35, 38, 40, 41, 44, 45, 46, 48, 51] the EVOO (Extra Virgin Olive Oil) was considered an important element of MD. In 2 studies [26, 27], some mobile devices (apps) were employed in the research design. The duration of the studies ranged from 2 weeks to 5 years according to the aim, design and study population.

### 3.3 Effectiveness of MD in Healthy Subjects

A total amount of 4092 healthy subjects were recruited in 5 studies. Gonzales Sanchez *et al*. [26] reported on the effect on cardiovascular risk factors (CVRFs) of an intervention based on the use of a smartphone app associated with a consultation on physical activity and MD for 3 months and follow up at 12 months. The main result was the reduction of CVRFs such as systolic blood pressure, diastolic blood pressure, total cholesterol and triglycerides. The use of the mobile device for three months compared to standard diet and physical activity counseling did not add any further benefit in terms of reduction of CVRFs. Hurtado-Barroso et.al [28] reported on the reduction of vascular biomarkers after 2 weeks of polyphenol-rich diet among 22 healthy men. Subjects were randomly assigned to their usual diet *versus* a diet rich in polyphenols or low antioxidant diet. Polyphenols in urine were dosed as a compliance marker. Nitric oxide levels were also measured as biomarker and it was of note that slight dietary modifications (such as adjunctive foods rich in polyphenols) are associated with some variation in vascular biomarkers. In 84 healthy people [32], 60.06 ± 6.93 years old, two interventions (for 12 weeks) based on home visits with strength exercises and nutritional topics *versus* a control group receiving home visits with social support, have been compared. There were no significant changes in physical activity, quality of life and anthropometric measurements among the groups probably suggesting that the involvement of over 60 years old health volunteers in the study may lead to some limited health benefits. Gomez *et al*. [33] employed the Thao-Child Health Program (TCHP), defined as a community-based intervention for healthy weight development and lifestyle choices, involving 2086 school-aged children. The program included seminars on eating habits and cooking techniques, physical activity, conferences, sport community days, etc., compared to the usual health policy for the control group. The intervention did not report short-term benefits for children with obesity. 1607 healthy subjects [39] were exposed to a web-based nutritional intervention (named *Food4Me*) providing a collection of dietary, anthropometric and biochemical data from volunteers receiving personalized nutritional advice *versus* general healthy eating information. After a 6 months period, volunteers with a low genetic risk score for metabolic issues reported a greater reduction in total cholesterol whereas those with high baseline MD score reported a great decrease in Body Mass Index (BMI), waist circumference and fasting glucose. The results of this study suggested that adherence to MD may lead to beneficial effects on metabolic outcomes, which may be influenced by the personal genetic risk score.

### 3.4 Effectiveness of MD in Elderly Subjects

The impact of MD on elderly subjects was tested by Jennings *et al*. [34] who described variations of inflammatory indexes, bone mineral density (BMD) and biomarkers of bone and collagen degradation in a sample of 1294 people. The MD employment did not show significant changes in BMD as well as urinary biomarkers. However, a subgroup analysis of individuals with osteoporosis at baseline (BMD T-score ≤ −2.5 SDs) showed that the MD may reduce the expected wane in femoral neck BMD (p = 0.04) but had no effect on the lumbar spine or whole-body BMD. Davis *et al*. [42] also reported that MD reduced triglycerides after 3 months (p< 0.001) and at 6 months (p = 0.03) and reduced also F2-IsoPs (F2-isoprostanes; as lipid biomarkers for oxidative stress) after 3 months (p < 0.001) and 6 months (p < 0.001). Lipoproteins, glucose, insulin, and protein- c reactive concentrations were not significantly modified by MD.

### 3.5 Effects of MD on Pregnant and Post-menopausal Women

Vignini *et al*. [40] tested if dietary supplementation with extra-virgin olive oil (VOO) enriched with vitamins D3, K1, and B6 (VitVOO) could modify the expression of stress markers among 60 postmenopausal women. After 1 year, those women with a higher intake of VitVOO showed lower plasma stress markers levels. It should be noticed that, in the study design, the diet regimen was inspired by MD although no specific MD intervention has been employed.

Assaf-Balut*et al*. [38] described the impact of MD on the incidence of Gestational Diabetes Mellitus in two groups of pregnant women: 500 normoglycemic pregnant women at 8-12 gestational weeks were assigned to the intervention group based on integrated MD with extra virgin olive oil (EVOO) and pistachios or to the Control Group (n = 500) based on a standard diet with limited fat intake. The primary outcome was to evaluate the effect of the intervention on the incidence of gestational diabetes mellitus (GDM) at 24-28 gestational weeks. 177 out of 874 women, who completed the study, were diagnosed with GDM, 103/440 (23.4%) in the control group, and 74/434 (17.1%) in the intervention group (p = 0.012). MD also improved the outcome of GDM treated with insulin and reduced the possibility of perinatal trauma (*all* p <0.05). These data suggest that an integrated MD reduces the incidence of GDM and improves several maternal and neonatal outcomes.

### 3.6 Effectiveness of MD on Cardiovascular Diseases

The positive impact of MD on cardiovascular diseases has been widely explored and described in the last decade [11, 12].

Wade *et al*. [29] in the MedDairy randomized controlled trial tested the effect of MD on cardiovascular risk factors among 41 people at risk of cardiovascular disease. Group on MD reported lower morning systolic blood pressure (mean difference: −1.6 mm Hg; −0.4 mm Hg; p = 0.01), diastolic one (mean difference: −1.0; 95%, −0.2 mm Hg; p = 0.01), significantly higher HDL cholesterol, lower triglycerides and lower ratio of total to HDL cholesterol (0.0001> p < 0.01). Arpón *et al*. [35] explored methylation changes in genes of peripheral white blood cells (PWBCs) from participants in the PREDIMED-Navarra trial among 36 people with high cardiovascular risk addressed to two different dietary Mediterranean profiles. One of two methylated sites selected was associated with polyunsaturated fatty acids intake, showing a role for specific fatty acids on epigenetic modulation. They concluded that specific components of MD, particularly nuts and EVOO, may induce methylation changes in some specific PWBCs genes. Grimaldi *et al*. [36] tested the efficacy and the drop-out rate of a 3-month dietary intervention based on MD (including a subgroup of diabetic patients) for body weight and cardiometabolic risk factors among 116 reporting CV risk. After the intervention, body weight decreased significantly in MD and control groups (p < 0.001) even if the intervention group showed a significant higher reduction in weight, plasma glucose, triglycerides and blood pressure and an increase in HDL cholesterol than the control group. Also, in the subgroup of participants with type-2 diabetes mellitus, there was a significant improvement in glycosylated haemoglobin levels with intensive intervention (p < 0.0001).

Guasch-Ferré *et al* [41] interpreted results from PREDIMED on health benefits of the MD among 7477 subjects with high cardiovascular risk. PREDIMED reported that MD reduces the incidence of major cardiovascular events by approximately 30%. Other benefits may include positive effects on metabolic syndrome, obesity, cognition, and breast cancer as well as a significant reduction by 30% of diabetes complications such as retinopathy. A reduction of 66% of peripheral arterial disease in the MD plus EVOO group and 50% reduction in the MD plus nuts as well as a reduction of 38% of atrial fibrillation risk during the MD plus EVOO have been reported.

Estruch *et al*. [44] evaluated the long-term effects of high-fat diet *versus* high-vegetable-fat MD on weight and waist circumference among 7477 people at risk of cardiovascular disease recruited from PERDIMED- trial. After 5 years of observation, participants have shown a partial reduction of body weight and waist circumference: –0.43 kg bodyweight in the olive oil group (p=0.044) and –0.08 kg in the nut group (p=0.730); –0.55 cm of waist circumference in the olive oil group (p=0.048) and –0.94 cm (p=0,006) in the nut group. Also, Eguaras *et al*. [48] confirmed that MD may reduce the risk of cardiovascular disease in 7477 subjects (PREDIMED) with a direct impact on abdominal obesity and central adiposity. This was confirmed by the evidence of a significant reduction of Body Mass Index (p= 0.01) and waist circumference (p =0.043) over the 5 years of follow-up.

Gomez-Delgado *et al*. [46] examined the effect of two healthy dietary programs (MD with EVOO and low-fat diet) on the nucleotide polymorphisms (SNPs) of the CLOCK gene involved in the lipid metabolism and inflammation balance among 897 patients with a history of Chronic Hearth Disease. They observed a significant gene-diet interaction. Specifically, C/C allele carriers displayed a greater decrease in high sensitivity C-reactive protein (p < 0.001) and a significant increase in HDL/apolipoprotein A1 ratio (p = 0.029) than minor G allele carriers (G/G + C/G). Vázquez-Fresno *et al*. [51] assessed the effect of an MD on urinary metabolites in a sample of 98 non-diabetic men and women with high cardiovascular risk at 1 and 3 years of follow-up, after an MD supplemented with extra-virgin olive oil (MD + EVOO) or nuts (MD + Nuts) *versus* control group with a low-fat diet (LFD). LFD-associated metabolites were hippurate and metabolites related to histidine metabolism, as well as xanthosine. MD groups showed a moderate association with metabolites related to a higher intake of fruit and vegetables. Furthermore, a significant but moderate association with meat intake urinary metabolites has been observed in the LFD group.

### 3.7 MD Effect in Patients Affected by type-2 Diabetes Mellitus

Alonso-Domínguez *et al*. [27] in the EMID- study (Effectiveness of a Multifactorial Intervention in Diabetics) evaluated the effectiveness of a multifactorial intervention in increasing adherence to the MD, the quality of diet and biomedical parameters after a 12-month follow-up period. The study involved 204 subjects with age ranging from 25- 70 years old affected by type-2 diabetes mellitus. Participants were randomized to the intervention group (GI) and the control group (CG), both receiving advice on a healthy diet and physical activity. They also joined some seminars, 5 healthy walks and had to use a smartphone app on diet and a healthy lifestyle for three months. At the 3-month follow-up, there was an improvement in adherence to the MD and quality of the diet in the GI. This trend was confirmed at 12-month of follow-up.

Di Onofrio *et al*. [30] enrolled a total of 69 patients who joined a motivational program focused on MD, nutrient classes, distribution of meals during the day, and dietary choices. Clinical and metabolic parameters were also analyzed for evaluating the effect of the program. At the end of the intervention, the total daily consumption of kilocalories was reduced as well as the percentages of carbohydrates, proteins, and lipids. A significant improvement in systolic and diastolic blood pressure, BMI and waist circumference, as well as in blood glucose values was observed. This longitudinal study concluded that nutritional motivational interventions can be useful to improve eating habits, especially among patients affected by type 2 diabetes.

Monlezun *et al*. [47] provided medical school-based teaching about kitchen and MD - cooking for patients with type 2 diabetes by assigning patients between the Goldring Center for Culinary Medicine group and the control group. Compared to the control group, the GCCM group had a greater reduction in glycosylated hemoglobin (-0.4%; p = 0.575) even if not statistically significant. There was a significant reduction in the GCCM group of DBP (p = 0.037) and total cholesterol (p = 0.044). This is considered to be the first known RCT that demonstrated improvements in biometrics using a new MD-based intervention for diabetic patients.

### 3.8 Effects of MD on Metabolic Syndrome

In the study by Gomez-Huelgas *et al*. [49], 601 subjects with metabolic syndrome received an intensive lifestyle intervention (LSI) based on instructions on MD and regular physical exercise. The primary outcome included the reduction from baseline of anthropometric parameters like abdominal circumference, blood pressure, HDL cholesterol, fasting plasma glucose, and triglycerides. At the end of the intervention, the LSI- group reported significant differences compared to the control group in the abdominal circumference, systolic pressure, diastolic pressure, and HDL cholesterol; there were no significant differences in the fasting plasma glucose concentration and triglycerides. In a small study of Cases *et al*. [50], 17 obese volunteers (BMI: 30.1–33.3) were recruited in a double-blind, randomized, parallel pilot study based on a 900 mg/day polyphenol-rich treatment with extracts from fruits and vegetables for 12 weeks. Anthropometric and blood parameters were assessed before and at the end of the intervention period. After 12 weeks, metabolic parameters and overall satisfaction of patients were improved. These data suggest that the daily supplement of polyphenols and MD improves metabolic balance and quality of life of obese volunteers.

### 3.9 MD and Risk of Cancer

Zuniga *et al*. [31] conducted a study based on interventions for improving adherence to an MD/ anti-inflammatory dietary model in breast cancer survivors (BCS). Overweight and obese BCS were randomized to the intervention group (n = 76) or control group (n = **77**). The 6-month intervention included monthly seminars on food- selection and healthy-cooking, motivational phone calls. Control- group participants received monthly information brochures but no navigation services. Adherence to MD increased significantly in the intervention group over time, but not in the control group (+ 22.5% *versus* + 2.7%; p <0.001). Also, the intervention arm increased the use of spices and herbs compared to the control (+ 146.2% *versus* + 33.3%; p <0.001). This study may suggest that educational interventions in BCS lead to an increased adherence to Mediterranean-style dietary pattern, increasing the consumption of anti-inflammatory foods, with the reduction of pro-inflammatory foods selection.

Griffin *et al*. [25] tested the use of MD in reducing Trimethylamine N-oxide (TMAO) plasma concentrations among 115 healthy people at risk of colon cancer. Fasting TMAO concentrations were measured before and after 6 months of dietary intervention. No significant differences were found in plasma TMAO between the Mediterranean group and the healthy diet group. However, TMAO concentrations showed a positive correlation with age and metabolic markers. This finding may suggest that an extensive dietary intervention over 6 months may not be fully useful to reduce TMAO concentrations in a healthy population at risk of colon cancer.

Toledo *et al*. [45] evaluated the combined effect of MD versus general nutritional advice on the incidence of breast cancer among 4282 women with high cardiovascular risk. After a 5-years period of follow-up, 35 confirmed cases of breast cancer were found with lower rate in the MD with extra-virgin olive oil group (1.1%; hazard ratio [HR] *vs* the control group 0.32 [95%CI, 0.13-0.79]) < MD with nuts group (1.8%; HR 0.59 [95% CI, 0.26-1.^35^] < control group (2.9%).

### 3.10 MD and Mental Health

Parletta *et al*. [37] tested the effects of MD supplemented with fish oil on mental health among 152 people self-reporting depressive symptoms (30.9% males). The intervention group received MD-cooking workshops for 3 months and fish oil supplements for 6 months. 95 people completed assessments at 3-months and 85 people completed at 6-months. At 3 months, the MD group reported higher MD adherence (p < 0.01), consumed more vegetables (p < 0.01), fruit (p = 0.04), nuts (p = 0.02), legumes (p = 0.02) wholegrain (p = 0.01), and vegetable (p< 0.01); less unhealthy snacks (p = 0.04) and red meat/chicken (p = 0.04). The MD group also reported a greater reduction in depressive symptoms (p= 0.03) and improved mental health QoL (Quality of Life) scores (p= 0.04) at 3 months. Improved diet and mental health improvements were confirmed at 6 months. In addition, reduced depression was significantly correlated with an increased MD – adherence (p = 0.01), more consumption of nuts (p = 0.01), and vegetable (p= 0.01). Mental health (QoL) improvements significantly correlated with increased vegetable and legumes consumption. Also, some expected positive correlations between increased omega-3, decreased omega-6 and improved mental health were found.

Jacka *et al*. [43] investigated the efficacy of a dietary program based on MD for the treatment of symptoms related to major depressive episodes in 67 subjects (72% female) with Major Depressive Disorder (according to DSM-IV-R). MD group was compared to a social support control group, both observed for 12 weeks. The MD group demonstrated significantly greater improvement at Montgomery–Åsberg Depression Rating Scale (MADRS) than the social support control group, (p < 0.001). Remission (defined as MADRS score <10) was obtained for 32.3% (intervention group; n = 10) and 8.0% (control group; n = 2) subjects respectively (p = 0.028); number needed to treat (NNT) based on remission scores was 4.

Both studies available seem to suggest significant benefits in employing MD-regimen among patients reporting depressive symptoms, in terms of reduction of symptoms over time and improved rates or remission.

## 4 DISCUSSION AND CONCLUSION

The main challenges in the study of the role of MD in health and mental health are related to the accurate assessment of exposure to dietary models, the heterogeneity of populations involved and the comparison between clinical and non-clinical conditions that could benefit from a dietary intervention [10]. This mini-review aimed to synthesize the existing literature based on clinical trials, excluding review articles, case reports or study protocols, and report on findings regarding MD, physical health and mental health.

It is of note that MD has a confirmed role in improving metabolic as well as cardiovascular parameters with a reduced incidence of major cardiovascular events by approximately 30% [41]. MD also has benefits in improving metabolic balance in patients affected by type 2 diabetes mellitus [27, 30]. The improvement of biochemical markers has been documented for metabolic disorders as well as in patients at risk of specific cancer diseases [25]. Dietary regimens based on polyphenols, fibers, EVOO or nuts may greatly reduce oxidative biochemical processes on the base of metabolic, cardiovascular and cancer pathophysiology [2, 3, 5].

The role of diet, and in particular MD, in the development of mental disorders has become a recent research focus over the past decade [18, 22]. Even if the efficacy of dietary programs in the management of metabolic issues among patients affected by severe mental illness is well known and described [21, 22, 23], few data are available on the MD effects on specific psychopathological issues. Two recent clinical trials [37, 43] about MD and mental health showed positive findings on the improvement of depressive symptoms and remission rates under a healthy diet regimen. Also, in 2020, a review of literature based on 37 studies confirmed an association between polyphenols consumption and depression risk, as well as a reduction of depressive symptoms severity [52]. Besides, adherence to MD is a key factor for promoting outcome improvements in depressive patients, as suggested by an observational study reporting an inverse association between adherence and severity of symptoms as well as poor depressive outcomes among patients with comorbid overweight or metabolic syndrome [53].

This review suggests further studies to determine the role of diet, MD, specific nutrients, and dietary programs in the outcomes of physical diseases and mental disorders. These may also add evidence on the pathophysiology and treatment of illness in the framework of the *Gene x Environment* interaction. Specifically, focused experimental protocols should address the efficacy of diet as an adjunctive treatment for mental disorders as well as for the management of comorbid cardiovascular and metabolic issues [54].

## Figures and Tables

**Fig. (1):**
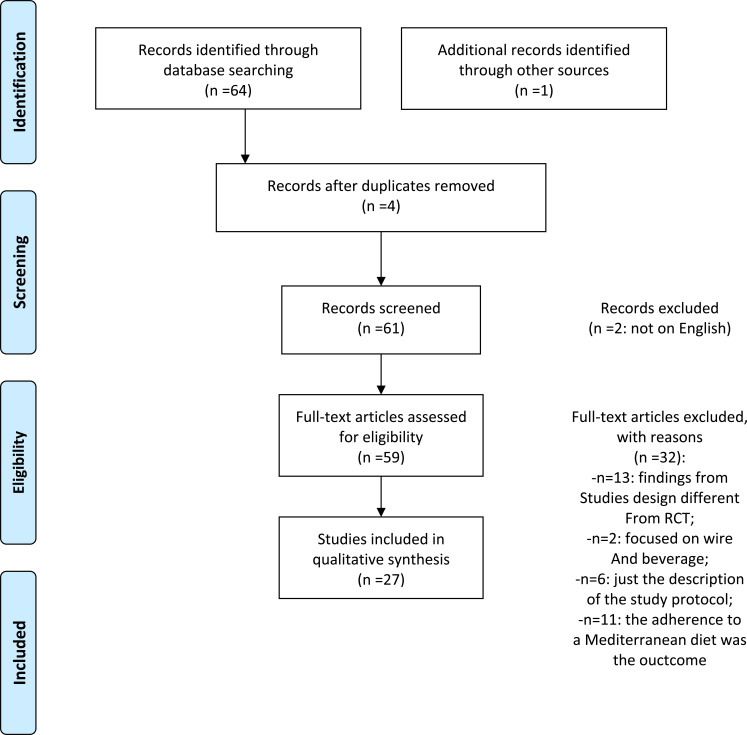
Studies selection (PRISMA).

**Table 1:** Studies population.

**Study Population**	**Number of Studies**	**Reference #**	**Population Involved** **(subjects #)**
**Healthy or Young Subjects**	5	[26, 28, 32, 33, 39]	4092
**Post-menopausal Women**	1	[40]	60
**Elderly Subjects**	2	[34, 42]	1460
**Pregnant Women**	1	[38]	1000
**Depressive Disorder Patients**	2	[37, 43]	219
**Type 2- Diabetes Mellitus**	3	[27, 30, 47]	300
**Cardio-vascular Diseases**	9	[29, 35, 36, 41, 44, 45, 46, 48, 51]	8531
**Cancer**	2	[25, 31]	268
**Metabolic Syndrome**	2	[41, 49]	618

**Table 2:** Age of subjects involved in 27 studies.

**Study Population**	**Number of Studies**	**Reference #**	**Mean Age of Subjects Involved (without standard deviations)**
**Healthy or Young Subjects**	5	[26, 28, 32, 33, 39]	27.63
**Post-menopausal Women**	1	[40]	55.6
**Elderly Subjects**	2	[34, 42]	70.91
**Pregnant Women**	1	[38]	32.7
**Depressive Disorder Patients**	2	[37, 43]	44.32
**Type 2- Diabetes Mellitus**	3	[27, 30, 47]	61.5
**Cardio-vascular Diseases**	9	[29, 35, 36, 41, 44, 45, 46, 48, 51]	64.8
**Cancer**	2	[25, 31]	54.7
**Metabolic Syndrome**	2	[41, 49]	53.4

**Table 3:** Body Mass Index of subjects of studies.

**Study (reference #)**	**BMI (kg/m^2^; mean ± standard deviation)**
[25]	27.0 ± 3.7
[26]	28.06 ± 5.12 (intervention); 27.57 ± 4.59 (control)
[27]	29.5±4.2 (intervention); 30.3±5.6 (control)
[28]	24.8±0.79 (intervention); 24.9±0.79 (control)
[29]	30.8 ± 3.8
[30]	31.26±9.47
[31]	31.2 ± 4.1 (intervention); 32.7 ± 5.2 (control)
[32]	25.64±5.39
[33]	18.8±3.2 (intervention); 18.6±3.0 (control)
[34]	26.9 ± 4.2 (intervention); 26.7 ± 3.8 (control)
[35]	27.3±3.2 (control); 27.9±1.5 (first arm); 28.1±1.5 (second arm)
[36]	32.3 ± 4.2 (intervention) 32.6 ± 3.9 (control);
[37]	not reported
[38]	not reported
[39]	25.4±4.7
[40]	25.9 ± 3.1 (intervention) 25.2 ± 2.7 (control)
[41]	not reported
[42]	74.0 ± 13.0 (intervention) 75.4 ± 12.9 (control)
[43]	29.5 ± 8.0
[44]	30.2 ± 4.0 (control) 29.9 ± 3.7(first arm) 29.7 ± 3.8 (second arm)
[45]	30.4 ±3.9 (control) 30.2 ±4.1(first arm) 30.7 ±4.2(second arm)
[46]	not reported
[47]	37 (intervention: range 32–41); 36 (control: range 30-42)
[48]	29.9±3.7 (first arm); 29.7±3.8 (second arm); 30.2±4.0 (control)
[49]	31.1 ± 4.72
[50]	30 – 34.9
[51]	reported in supporting information 30.2±3.8
