# Mediterranean Diet and its Benefits on Health and Mental Health: A Literature Review

**DOI:** 10.2174/1745017902117010009

**Published:** 2021-03-26

**Authors:** Antonio Ventriglio, Federica Sancassiani, Maria Paola Contu, Mariateresa Latorre, Melanie Di Salvatore, Michele Fornaro, Dinesh Bhugra

**Affiliations:** 1 Department of Clinical and Experimental Medicine, University of Foggia, Foggia, Italy; 2 Dipartimento di Scienze Mediche e Sanità Pubblica, Università degli Studi di Cagliari, Cagliari, Italy; 3 Dipartimento di Scienze Chirurgiche, Università degli Studi di Cagliari, Cagliari, Italy; 4 Department of Neuroscience, Reproductive Science and Odontostomatology, School of Medicine 'Federico II' Naples, Naples, Italy.; 5 Institute of Psychiatry, King’s College, London, UK

**Keywords:** Mediterranean Diet, Health, Mental Health, Depression, Anxiety, Cancer

## Abstract

Mediterranean Diet (MD) is currently considered one of the most healthy dietary models worldwide. It is generally based on the daily intake of fruit and vegetables, whole grains, legumes, nuts, fish, white meats, and olive oil. It may also include moderate consumption of fermented dairy products, a low intake of red meat, and red/white wine during the main course. Even if the effect of MD on cancer prevention as well as on human metabolic and cardiovascular balance has been discussed, including the quality of life of the exposed population, the putative effects on mental health are still not properly investigated. This narrative review reports on some emerging pieces of evidence on the possible impact of MD on general health and the outcome of psychiatric disorders (*e.g*., major depression, anxiety) and encourages further studies to test the benefits of healthy food selection on the health of the general population.

## CORRIGENDUM

Mediterranean Diet and its Benefits on Health and Mental Health: A Literature Review Clinical Practice & Epidemiology in Mental Health, 2020, 16: 156-164 

## Correction

The corrections are provided and replaced online which is mentioned as under:

Original:

The name of coauthor was Melanie Di Slavatore

Corrected:

The name of coauthor has been revised as Melanie Di Salvatore

